# Editorial: Falls prevention for older people in primary care settings

**DOI:** 10.3389/fpubh.2022.1106121

**Published:** 2022-12-21

**Authors:** Lynette Mackenzie

**Affiliations:** Discipline of Occupational Therapy, School of Health Sciences, Faculty of Medicine and Health, The University of Sydney, Sydney, NSW, Australia

**Keywords:** accidental falls (MeSH), primary health, aging, interventions, environment

## Introduction

As our populations are aging globally, there is an imperative to ensure older people can age as healthy and active participants as far as possible. Falls and falls-related injury are likely to be major health care problems for older people as they age for the foreseeable future. The identification and implementation of effective falls prevention strategies is an important public health priority, and many such strategies will need to take place in the primary care environment.

Currently, known effective interventions include balance and strength exercise, expedited cataract surgery, podiatric interventions, occupational therapy interventions, and targeted multi-factorial interventions. Greater understanding is required regarding optimal approaches to multi-factorial interventions, fall prevention in hospital and residential care, behavior change strategies, cost-effectiveness of fall prevention interventions, and fall-injury prevention.

This Research Topic includes a broad collection of contributions focused on the full range of primary health strategies to address increasing falls risk for older people. Research Topics include how to market balance workshops for fall prevention to older people, environmental issues such as the story of the home and the reliability of a home environmental screening tool for falls risk, the walkability of urban settings for older people, the role of physical therapists in identifying older people at risk and referring them to community programs, the identification if single fallers compared to recurrent fallers, issues of the fear of ambulating among residents of long term care facilities and screening for foot and footwear risk factors for falls. Contributors are from Europe, Asia, North and South America reflecting a global interest in this Research Topic.

We will briefly overview the articles in this Research Topic according to three main themes: (i) person related falls risk factors, (ii) environmental issues related to falls prevention in primary care, and (iii) potential screening and intervention strategies.

## Themes

### Person-related falls risk factors

Older people are a very diverse group and will present with a diverse range of falls risk factors relating to their individual functional changes and limitations and their personal beliefs and behaviors in relation to falls risk. Górski et al. described ambulophobia—a fear of walking, which takes the form of older people not to leaving their bed or home to avoid walking on uneven surfaces and reduce the risk of falling. They identified 30% of older people in long term care facilities presenting with this phobia. Ambulophobia was associated with being female, aged 71–80 years, depression, Parkinson's disease, orthostatic hypotonia, a history of falling or seeing another person fall, polypharmacy and moderate or severe disability in activities of daily living. All of these factors are also fall risk factors and falls most frequently occur during mobility, therefore, fears of walking are not unreasonable in these circumstances. For people living in long term care facilities these risk factors are prevalent suggesting ambulophobia needs to be considered amongst residents.

In contrast Suzuki et al. investigated the walking experiences of older adults with functional limitations living in the community. They found that older people with functional limitations were less likely to visit places within walking distance. Many local environments were not well-lit at night and had more walking hazards. Older people's perceptions of walkability were also important irrespective of functional limitations. Strategies are needed to encourage walking for older people especially in lower-income residential communities.

### Managing fall prevention

Papers in this theme were focused on the assessments used to evaluate risks of falls, how information about falls prevention interventions is disseminated within the community, and the role of one health profession in screening for falls risk and referring on to community programs.

Ferreira et al. evaluated the reliability of the HOME FAST BRAZIL—self report version (Home Falls and Accidents Screening Tool—Brazil) with 50 community dwelling older people in Brazil. The tool was applied by two raters (evaluator 1 and evaluator 2) to analyze inter-rater reliability on the same day, independently, with an interval of 40 min. The intra-rater reliability was determined by applying the tool by the same evaluator (evaluator 1) on two different occasions, with an interval of 7 days between applications. Inter-rater and intra-reliability intra-class correlations were adequate. A risk of falls was verified in 88% of the sample and four environmental risks presented significant correlations with the history of falls (difficult armchairs transfers, the absence of anti-slip mats in the shower recess, the presence of pets and difficult bed transfers).

Another Brazilian study by da Silva et al. undertook an evaluation of seven assessments to determine cut-off scores that differentiated non-fallers, one-time fallers and recurrent fallers (two or more falls per year) in a cross-sectional retrospective study of 90 community dwelling pre-frail women. Recurrent fallers had lower usual gait speed and isometric peak torque of knee extensors than the one-time fallers, and lower fast gait speed than both one-time fallers and non-fallers. One-time fallers were differentiated from non-fallers by walking speed reserve. Recurrent fallers were differentiated from non-fallers by fast gait speed and step length. They concluded that gait speed, step length, peak torque of both knee extension and flexion, and ankle dorsiflexion can be used to identify both one-time and recurrent fallers.

The final assessment tool study was presented by Wingood et al. who developed the Screening Tool for Feet/Footwear-Related Influences on Fall Risk. Items were determined by an expert panel and a Delphi process, resulting in a 20-item tool. The tool can be used to screen for feet/footwear-related influences on fall risk among community-dwelling adults identified at risk of falling.

Looking at other management practices to promote falls prevention in the community, Vincenzo et al. described how physical therapists could be involved in community screening for falls prevention using a survey with 444 therapists in the United States. They concluded that because of their falls prevention screening behaviors physical therapists and physical therapy assistants are key partners in evidence-based multifactorial fall prevention in the community. Goethals et al. presented a protocol for a study to investigate social marketing in the French context to enable older people to access group balance workshops. The proposed campaign consists of using advertisements posted in the local print media, flyers for distribution by local partners and conferences for older people to reach the target audience.

### Environmental influences on falls prevention in the community

Tsai et al. presented the only paper directly addressing environmental falls risk factors. They examined the association between the story of the building that an older adult resided in and their fall risk in their residences and their level of fear of falling using US data. They used large samples of 6,153 for falling and 6,142 for worry about falling. There was a higher prevalence of falls and worry about falling when participants lived in a single story building compared with participants who lived in a multi-story building, however logistic regression analysis showed no highly significant association between the story of building and falling or experiencing fear of falling.

Effective strategies are needed in primary care services to both screen for falls risk and manage falls prevention with older people living in the community. This Research Topic of papers has provided a range of evidence relating to what is effective in the primary care setting and can offer some guidance to health practitioners in primary health settings. More work is needed to fully operationalize falls prevention within primary health. One simple intervention is to ensure that older people who are seen by any health professional in primary care are asked about falling and can be referred to appropriate prevention services, long before the risk is severely elevated, or before a serious fall has occurred. This means educating all the relevant health professionals working in community and primary health settings so they can understand their role and the roles of others, and work together to reduce falls.

It is important to acknowledge the efforts of the Editorial team for this Research Topic who contributed their expertise in falls prevention to their reviews of submitted manuscripts.

## Author contributions

The author confirms being the sole contributor of this work and has approved it for publication.

